# The Vitamin D, Ionised Calcium and Parathyroid Hormone Axis of Cerebral Capillary Function: Therapeutic Considerations for Vascular-Based Neurodegenerative Disorders

**DOI:** 10.1371/journal.pone.0125504

**Published:** 2015-04-13

**Authors:** Virginie Lam, Ryusuke Takechi, Menuka Pallabage-Gamarallage, Corey Giles, John C. L. Mamo

**Affiliations:** 1 School of Public Health, Faculty of Health Sciences, Curtin University, Perth, Western Australia, Australia; 2 Curtin Health Innovation Research Institute of Ageing and Chronic Disease, Curtin University, Perth, Western Australia, Australia; Oklahoma State University, UNITED STATES

## Abstract

Blood-brain barrier dysfunction characterised by brain parenchymal extravasation of plasma proteins may contribute to risk of neurodegenerative disorders, however the mechanisms for increased capillary permeability are not understood. Increasing evidence suggests vitamin D confers central nervous system benefits and there is increasing demand for vitamin D supplementation. Vitamin D may influence the CNS via modulation of capillary function, however such effects may be indirect as it has a central role in maintaining calcium homeostasis, in concert with calcium regulatory hormones. This study utilised an integrated approach and investigated the effects of vitamin D supplementation, parathyroid tissue ablation (PTX), or exogenous infusion of parathyroid hormone (PTH) on cerebral capillary integrity. Parenchymal extravasation of immunoglobulin G (IgG) was used as a marker of cerebral capillary permeability. In C57BL/6J mice and Sprague Dawley rats, dietary vitamin D was associated with exaggerated abundance of IgG within cerebral cortex (CTX) and hippocampal formation (HPF). Vitamin D was also associated with increased plasma ionised calcium (iCa) and decreased PTH. A response to dose was suggested and parenchymal effects persisted for up to 24 weeks. Ablation of parathyroid glands increased CTX- and HPF-IgG abundance concomitant with a reduction in plasma iCa. With the provision of PTH, iCa levels increased, however the PTH treated animals did not show increased cerebral permeability. Vitamin D supplemented groups and rats with PTH-tissue ablation showed modestly increased parenchymal abundance of glial-fibrillary acidic protein (GFAP), a marker of astroglial activation. PTH infusion attenuated GFAP abundance. The findings suggest that vitamin D can compromise capillary integrity via a mechanism that is independent of calcium homeostasis. The effects of exogenous vitamin D supplementation on capillary function and in the context of prevention of vascular neurodegenerative conditions should be considered in the context of synergistic effects with calcium modulating hormones.

## Introduction

In primary neurodegenerative conditions such as vascular-dementia, Alzheimer’s disease, multiple sclerosis and epilepsy; and in secondary neurodegenerative disorders such as stroke, cerebral capillary function is impaired, resulting in inappropriate blood-to-brain parenchyme protein trafficking, neurovascular inflammation and if persistently exaggerated, cellular apoptosis [1, 2]. Consistent with a causal association of capillary dysfunction in some neurodegenerative disorders, there is an accumulating body of literature from clinical studies and in animal models showing therapeutic benefit in disease progression if blood-brain barrier (BBB) disturbances are corrected or attenuated [3, 4].

Epidemiological and experimental studies suggest that vitamin D generally has a protective role in neurodegenerative disorders although the mechanism(s) for these purported benefits are not clear [5–7]. Analyses of transcription factors and signaling pathways support the contention that a key effect of vitamin D is via positive regulation of genes broadly involved in neurovascular inflammation [8]. However, other mechanisms relevant to neurodegenerative conditions may include modulation of p-glycoprotein expression; up-regulation of serotonin synthesizing genes; protein oligomerization (such as beta-amyloid) and apoptosis [9–11]. Some reported positive downstream effects of vitamin D include restoration of capillary function in models of multiple sclerosis and cessation of disease progression; enhanced cognitive performance in subjects with mild cognitive impairment; decreased risk for Alzheimer’s disease; and in some psychiatric disorders, an improvement in behavior [12–17].

A consequence of the perceived positive effects of vitamin D in reducing risk of neurodegenerative disorders has generated momentum in developed nations with aging populations, to consider adopting policies that promote vitamin D supplementation. However, this is a contentious issue, as robust physiological studies to investigate potential vitamin D toxicology are not yet realized and the ‘optimal’ vitamin D level remains controversial [5–7]. Moreover, the effects of vitamin D may be via regulation of calcium homeostasis, or the effect of calcium regulatory hormones, rather than via direct effects of the vitamin per se. Presently, there is little evidence to support the hypothesis that vitamin D at greater than ordinary physiological concentrations is likely to be beneficial.

An equivocal function of vitamin D is to regulate calcium metabolism. In response to low serum levels of ionised calcium (iCa), sensor cells within parathyroid tissue promote secretion of parathyroid hormone (PTH), which rapidly stimulates the conversion of vitamin D to its bioactive form 1,25-dihydroxyvitamin D_3_ (1,25(OH)_2_D_3_). Bioactive vitamin D and PTH will synergistically increase plasma iCa via enhancing intestinal absorption of dietary calcium, increasing osteoclast activity to liberate calcium and reducing renal calcium excretion. The provision of supplemental vitamin D progressively increases iCa and concomitantly suppress PTH secretion. Clearly then, cerebral capillary effects of exogenous vitamin D supplementation cannot be considered in isolation.

Calcium is pivotal to neuronal excitability and indeed the changes that underlie learning and memory [18–20]. In addition, calcium ions are critical in maintaining the integrity of the BBB [21, 22]. Several studies have implicated an association between elevated serum iCa and global cognitive decline [23–25]. Moreover, at greater concentrations and when chronically exaggerated, serum iCa is positively correlated with cerebral white matter lesions, greater lesion volume and induction of the apoptotic cascade [19, 26–29]. If iCa is central to the neurovascular effects of vitamin D, then by extension of the clinical observations, supplementation with vitamin D would notionally be beneficial in subjects with hypocalcemia, but possibly harmful in subjects with raised, or adequate levels of serum calcium. Tai et al reported that the influx of iCa into the CNS is not regulated by a saturable mechanism that is sensitive to acute changes in plasma concentration, but more likely to occur through passive diffusion [30]. The CNS would therefore notionally be protected from rapid acute changes in iCa [31, 32]. However, chronic heightened effects may be realized. The effects of iCa on capillary permeability and on neurovascular inflammation per se, have not been directly investigated.

Several lines of evidence also suggest that PTH may directly affect neurovascular integrity independent of vitamin D homeostasis. PTH has been shown to cross the BBB and an exaggerated level of PTH within CSF appears to cause neuronal degeneration due to cellular iCa overloading [33, 34]. In clinical studies, increased levels of PTH have been reported as an independent risk factor of age-related cognitive decline [35, 36]. Furthermore, case-controlled findings show improved cognitive performance in subjects with hyperparathyroidism following parathyroid surgery [37, 38]. Therefore, it is possible that studies reporting detrimental effects of vitamin D deficiency may be a surrogate marker of PTH induced sequelae.

Whilst capillary integrity is considered pivotal to risk or progression of a range of neurodegenerative disorders, regulation of capillary function via the vitamin D-iCa-PTH axis is neither established nor elucidated. However, delineation of the putative synergistic effects of vitamin D-iCa-PTH on capillary integrity may be of significant therapeutic importance. To this effect, this study adopted intervention models of dietary vitamin D supplementation; exogenous parathyroid hormone supplementation or parathyroid tissue ablation in two genetically unmanipulated rodent models, to investigate capillary permeability and neurovascular inflammation within CNS regions of interest that are relevant to learning and memory.

## Materials and Methods

### Animals and dietary/hormonal interventions

All animals were housed in accredited animal holding facilities in individual ventilated cages with 12-h light/dark cycle, controlled air temperature (21°C) and air pressure (Curtin Animal Holding Facility, Perth and Charles River Laboratories, Kent). All animals were given ad libitum access to specified diets and water. Dietary protocols and surgical procedures described in this study were approved by NHMRC accredited Curtin Animal Ethics Committee (approval no. N34-10 [mice] and AEC_2011_30A [rats]) and Charles River UK Ethics Committee (project Licence no. 70/7221), respectively. All surgical and endpoint protocols were performed under strict anaesthetic protocols to minimize pain and stress.

### Dietary Vitamin D (VD) supplementation

7-week-old female wild-type C57BL/6J mice and Sprague-Dawley rats were purchased from Animal Resources Centre (Murdoch, W.A, Australia). Animals were randomly assigned in groups of 8 to one of the following semi-purified A1N93G diets: Control 0.5% Calcium, 0.35% phosphorus containing 1000 IU; 20,000IU; 40,000 IU; 80,000 IU or 120, 000 IU vitamin D_3_/kg diet (Specialty Feeds, Glenn Forrest, W.A, Australia). Eight animals from each group were sacrificed at either 6, 12 or 24 weeks after commencement of dietary intervention. The approximate daily intake of VD per animal (international units) is listed in Table 1.

**Table 1:** Daily intakes of vitamin D.

Study	Vitamin D_3_ (VD) per kg of diet (IU)	Control (1, 000)	20, 000	40, 000	80, 000	120, 000
**Mice**	**Daily intake of VD (IU/day)**	2	40	80	160	240
	**Intake of VD/kg/day (IU)**	100	2, 000	4, 000	10, 000	16, 000
**Rats**	**Daily intake of VD (IU/day)**	15	300	600	1, 200	1, 800
	**Intake of VD/kg/day (IU)**	75	1, 500	3, 000	6, 000	9, 000

The approximate international units (IU) of vitamin D consumed by Sprague-Dawley rats and C57BL6/J mice per respective treatment group are shown as units consumed per day or daily units consumed per kilogram of weight.

### Parathyroid Gland Ablation

Seven week-old female Sprague-Dawley rats (200–250g) were randomly assigned as control animals or subjected to selective parathyroidectomy (PTX) (Charles River Laboratories, Kent, UK). Briefly, rats were placed under general anaesthesia via isofluorane and with the aid of a dissecting microscope; parathyroid glands were identified, dissected and ablated from the surrounding tissue. The skin wounds were closed using wound clips. Thereafter, animals were provided with 1.5% calcium lactate solution 3 days post-operation. Both control and PTX groups were given ad libitum access to standard SDS-VRF-1 diet (Calcium 1% Phosphate 0.6%) manufactured by Special Diet Services, UK and sacrificed at either 12 or 24 weeks. Successful parathyroid tissue ablation was confirmed by determining circulating serum PTH. The PTX treatment group data is indicative of rats with a PTH concentration <65pg/ml. At 12 weeks and 24 weeks post surgery data is presented for 4-PTX and 7-PTX rats, respectively. Eight controls were studied alongside each PTX treatment group, at 12 and 24 weeks.

### Exogenous Parathyroid Hormone Infusion

Seven week-old female Sprague-Dawley rats were randomly allocated to either control or PTH-infused groups. All animals were placed under general anaesthesia and Alzet mini osmotic pumps (2ML) were implanted subcutaneously between the scapulae of each animal (Charles River Laboratories, Kent, UK), containing either saline and 2% cysteine (control group) or rat 1–34 PTH fragment diluted in isosmotic saline with 2% cysteine at a concentration of 0.332ug/hour/rat (Bachem Labs, Switzerland). Skin wounds were closed using wound clips. All osmotic pumps were primed and filled under aseptic conditions. Animals were given ad libitum access to standard SDS-VRF-1 diet (Special Diet Services, UK) and sacrificed at either 6 (control n = 9; PTH- infused n = 8) or 12 weeks (control n = 8; PTH-infused n = 4). Treatment at 24 weeks could not be investigated as this would have required subsequent replacement of the mini osmotic pumps, a procedure not permitted by the ethics protocols. Rats with incomplete wound healing at one week post pump insertion were humanely euthanized under general anaesthesia. As exogenous PTH cannot be detected by PTH assays, we like other laboratories utilized serum measures of ionised calcium and total calcium as a surrogate marker of efficacy. In addition residual osmotic pump reservoir volume and weight confirmed delivery of the vehicle buffer/hormone.

### Sample collection and preparation

At each intervention endpoint, rats were anaesthetized with 75mg/kg ketamine and 10mg/kg xylazine whilst mice were anaesthetized with pentobarbitone (45 mg/kg) followed by exsanguination via cardiac puncture. An initial 100ul of fresh whole blood was collected into plain syringes for immediate analysis of blood ionised calcium whist the remaining sample was left to clot at room temperature for 30 minutes and centrifuged for serum extraction. Thereafter, samples were aliquoted and stored at -80°C until further analysis. Brain specimens were carefully extracted and fixed in 4% paraformaldehyde (w/v in PBS, pH 7.2) for 24h, followed by cryoprotection with 20% sucrose for 72 h at 4°C. The brain specimens were then frozen in dry ice/isopentane and stored at -80°C.

### Cerebral Extravasation of Immunoglobulin G

The integrity of cerebral capillaries was assessed by measuring cerebral perivascular extravasation of the plasma protein immunoglobulin G (IgG), a marker of unspecific blood-to-brain leakage of plasma proteins and macromolecules by 3-dimensional (3-D) semi-quantitative immunomicroscopy, using methods as previously described, with minor modifications [39]. Briefly, 20μm brain cryosections were blocked with 10% goat serum in PBS for 30 min and thereafter incubated with polyclonal goat anti-mouse IgG^1^ or goat anti-rat IgG^1^ antibodies conjugated with Alexa 488 fluorochrome (1:100; Invitrogen, USA) at 4°C for 20 h. Nuclear counterstains were performed for detection of cerebrovascular endothelial cells and the sections were mounted with anti-fade mounting medium. Negative controls were included with all the experiments, which included the replacement of the primary antibody with either buffer or an irrelevant serum. No fluorescent staining was observed in any of the negative controls.

Representative 3-D immunofluorescent micrographs were captured with AxioVert 200M (Carl-Zeiss, Germany) coupled with an mRM digital camera and ApoTome optical sectioning system. Each 3-D image was captured at a magnification of x200 (Plan-Neofluar 20x objective lens) and consisted of at least 12 2-dimensional Z- stack images with a 1.225μm axial distance optimized by Nyquist overlap theory. For each region of interest in the brain, a minimum of 10–30 3-D images was randomly captured from the cortex or hippocampal formation per animal. Capillary vessel fluorescence was excluded based on conservative threshold exclusion settings and confirmed manually for each image processed as previously indicated [4, 39, 40], avoiding confounders associated with extensive and incomplete perfusion with wash-out buffers. All 3-D images were used for the subsequent quantitative analysis. The voxel intensity of the fluorescent dye was calculated with Volocity 6.1 3-D image analysis software (PerkinElmer, UK) and expressed per volume unit. The average of the total fluorescent intensities of all the images in cortex and hippocampal formation regions were calculated within each animal and thereafter compared between each of the treatment groups.

### Immunofluorescent Analysis of Cerebral Inflammation

Similar to cerebral IgG immunomicroscopy, the cortex and hippocampal expression of glial fibrillary acidic protein (GFAP) was determined as a surrogate marker for astrocyte activation by utilizing immunodetection techniques as described previously [39, 41]. 20um brain cryosections were blocked with 10% goat serum and incubated with polyclonal rabbit anti-mouse GFAP (1:200; Abcam, UK) for 20 h at 4°C. Polyclonal goat anti-rabbit IgG conjugated with Alexa488 (1:200) was then applied to the sections for 2h at RT followed by DAPI nuclear counterstain. Slides were mounted with anti-fade medium. Negative controls were run with every experiment, which included the replacement of the primary antibody with either buffer or an irrelevant serum. No fluorescent staining was observed for control tissues with the indicated capture settings.

### Biochemistry Analyses

#### Blood Ionised Calcium

Serum ionised calcium levels were measured to determine the calcemic status of each animal. Whole blood was collected via cardiac puncture into non-coagulated syringes and immediately analysed with a hand-held VetScan iSTAT analyser and CG8+ blood gas cartridges (Abbott Point of Care, Australia) in accordance with the manufacturer’s instructions. Appropriate aqueous controls were run at every endpoint.

#### Serum Total Calcium and Inorganic Phosphate

Serum total calcium and phosphate were analysed by quantitative colorimetric assays by QuantiChrom Calcium and Phosphate Assay kits, respectively, according to manufacturer instructions (BioAssay Systems, CA).

#### Serum Parathyroid Hormone

Serum PTH was measured with ELISA kits specific for the detection of intact rat PTH 1–84 molecule (Immutopics Rat Intact PTH ELISA, San Clemente, CA). The kit is supplied with prepared standards and two control sera and has a sensitivity of 1.6pg/ml and range to 3000pg/ml for serum or plasma. Intra-assay precision of <2.4% and inter-assay variability of <6.0%. Reported values of PTH in rats have been reported between 40–400pg/ml [42–44]. In this study, serum was collected under identical conditions and PTH determined on the same day for all treatment groups indicated.

### Statistical Analysis

For quantitative immunomicroscopy, a minimum of 10 and up to 30 3-D images was captured from each mouse and rat per region of interest, respectively. In total over 800 (mice) and 4000 (rat) 2-D images were taken per group. All raw data was log transformed and the arithmetic mean was used as a measure of central tendency. Thereafter, all statistical analyses were done with non-parametric statistics by one-way analysis of variance (ANOVA) analyses followed by Tukey’s post hoc tests. Pearson’s correlation analysis was used to determine the association between parenchymal expression of IgG and specified plasma biomarkers. Data was considered to be statistical significant where p value <0.05. Unless otherwise stated, all data are expressed as mean +/ SEM. Statistical analyses were performed with IBM SPSS Version 22 (SPSS Inc., Chicago, USA).

## Results

**Fig 1:**
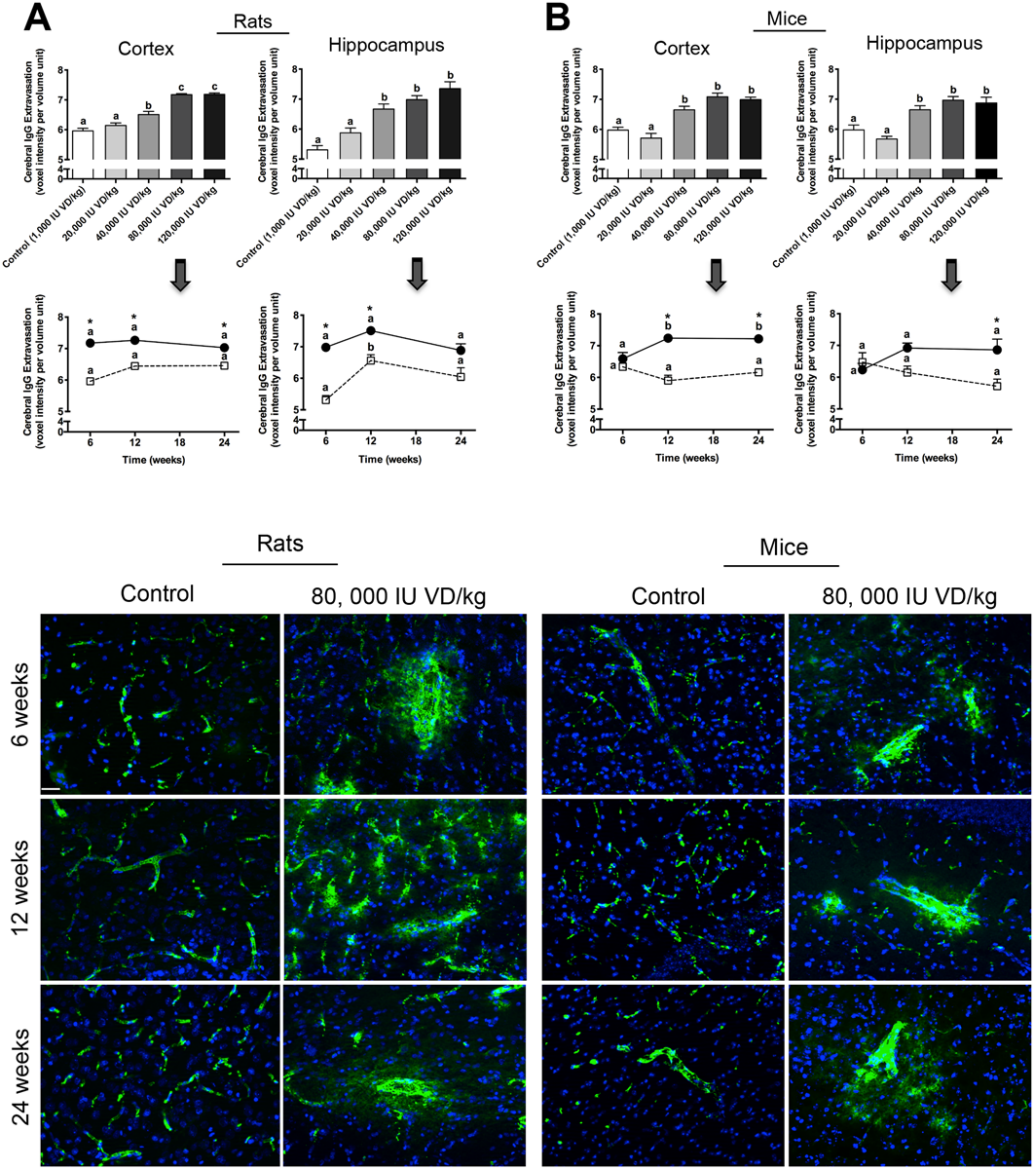
Cerebral Capillary Integrity. Cerebral capillary vessel integrity was assessed using 3-dimensional semi-quantitative fluorescent immunomicroscopy by measurement of parenchymal extravasation of plasma macromolecule IgG in the cerebral cortex (cortex) and hippocampal formation (hippocampus) of wild-type rats (A) and mice (B) supplemented with units of dietary vitamin D (VD) as indicated and measured in voxels per volume unit. The bar graphs illustrate a strong dosage effect of vitamin D concentration and abundance of cerebral IgG in the cortex and hippocampal formation in both species; statistical significance is denoted by different letters (p<0.05; n = 8; one way ANOVA followed by Tukey’s post hoc analysis). The bottom graphs demonstrate duration-of-treatment effects in both rodent species fed either control (open square/dotted lines) or diets enriched with 80, 000 IU VD/kg (black circles/solid lines) for 6, 12 and 24 weeks. Different letters show p<0.05 for duration effects within each group (n = 8) whilst an asterisk show statistically significant different dietary effects between control group and its’ relevant treatment group at each time point (p<0.05; n = 8; one-way ANOVA followed by Tukey’s post hoc analysis). Data is shown as mean ± SEM. Representative 2-dimensional extended-focus fluorescent immunomicrographs (magnification x 200) of cerebral IgG distribution in animals fed control and 80, 000 IU VD/kg diets are shown in the last frame; IgG is shown in green and DAPI nuclei counterstaining is shown in blue. Scale bar represents 100μm.

Parenchymal abundance of IgG was used to assess cerebral capillary permeability in wild-type mice and rats maintained on diets supplemented with vitamin D. Fig 1 depicts the abundance of IgG within cortex (CTX) and hippocampal formation (HPF) for the respective treatment groups. The findings show a strong positive association between parenchymal IgG within CTX and HPF and the dose of exogenous vitamin D provided. However, subtle differences in response to vitamin D supplementation were observed between Sprague-Dawley rats and C57BL/6J mice. Abundance of IgG within both CTX and HPF were markedly elevated within 6 weeks of treatment in rats consuming chow containing 80,000 IU VD/kg of diet, whereas the treatment effect was not seen in mice until approximately 12 weeks of feeding. Thereafter, both species showed stabilization in the abundance of parenchymal IgG, with persistently exaggerated amounts relative to control groups at 24 weeks of feeding, but comparable to vitamin D supplemented animals at 12 weeks of feeding.

**Fig 2:**
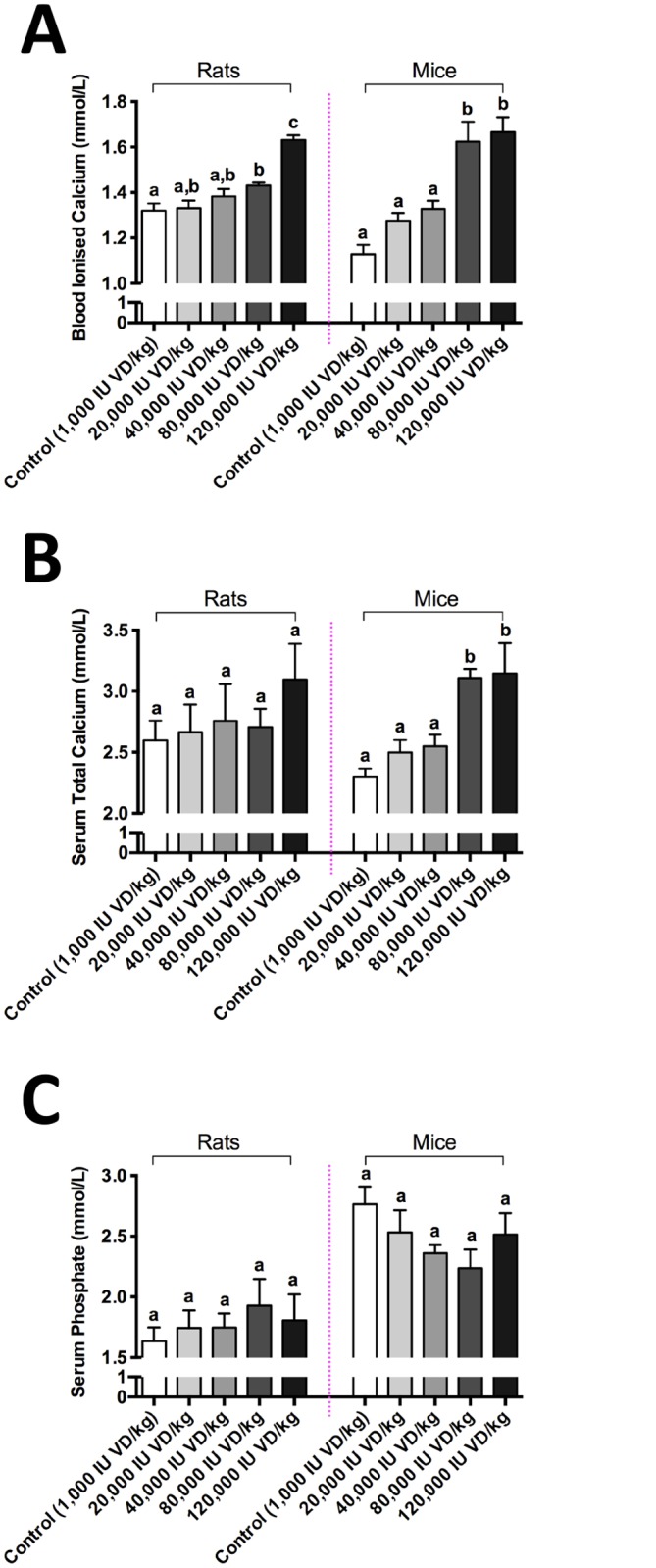
Blood biochemistry analyses. Blood biomarkers were measured in wild-type rats and mice supplemented with different doses of dietary vitamin D (VD) to assess calcemic status. Blood Ionised Calcium (B), serum Total Calcium (C) and Serum Phosphate were measured using either an iSTAT point-of-care analyser or commercially available colorimetric kits. Data is shown as mean ± SEM. Statistical significance is denoted by different letters (p<0.05; n = 8; one-way ANOVA followed by Tukey’s post hoc test).

**Fig 3:**
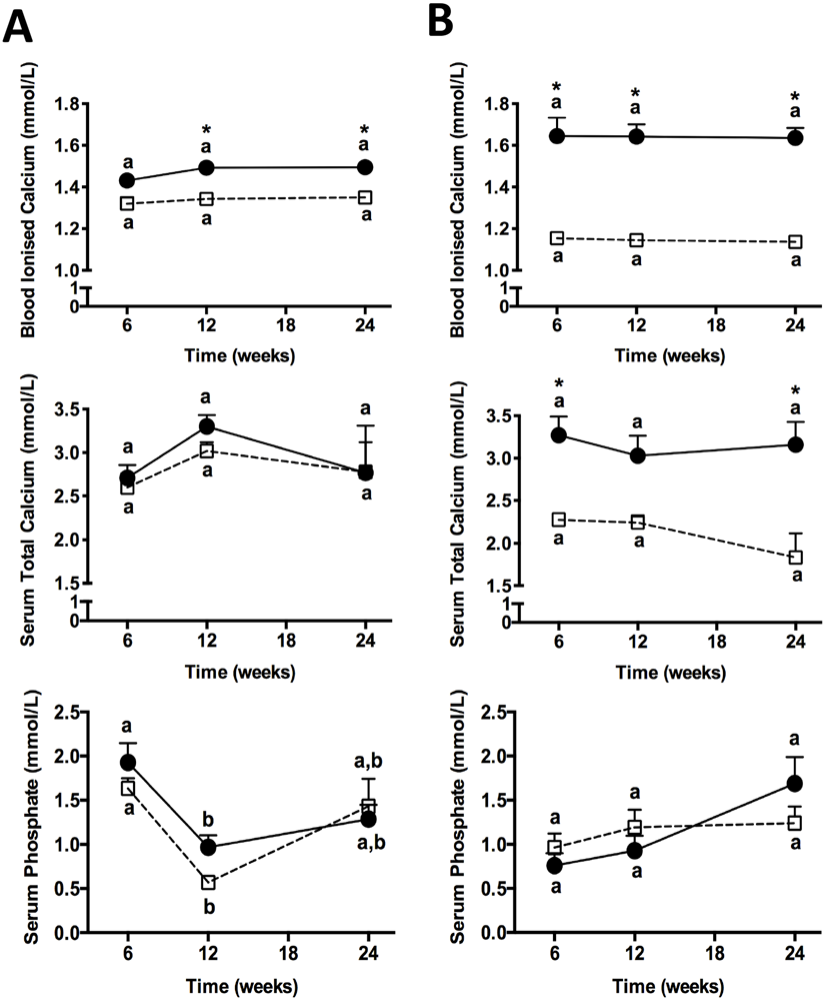
Duration-of-treatment effects of exogenous vitamin D on calcemic status. The duration-of-treatment effects of dietary vitamin D on blood ionised calcium, serum total calcium and serum phosphate in either control animals (open square/dotted lines) or animals fed 80, 000 IU vitamin D/kg (black circles/solid lines) at 6, 12 and 24 weeks of age are shown. Column (A) represents data derived from Sprague-Dawley rats and column (B) of C57BL/6J mice. p<0.05 for duration effects within each group is represented by different letters (n = 8; one-way ANOVA). * p<0.05 compared to relevant control at each corresponding time point (dietary effects; n = 8; one-way ANOVA). Data is shown as mean ± SEM.

Greater dosages of vitamin D in rats were required to increase serum iCa above controls compared to mice, however this may have reflected the markedly lower baseline values in control mice compared relative to control rats (Fig 2). Similarly, serum total Ca was progressively increased and phosphate decreased respectively in mice, whereas little change in rats was indicated at equivalent treatment dosages of vitamin D. A duration-of-treatment effect for the concentration of iCa, serum total calcium and phosphate is indicated in Fig 3. Vitamin D provided at 80,000IU VD/kg significantly increased bioactive iCa, compared to control group in mice within 6 weeks of treatment. However, effects were significant at 12 weeks for both species. Thereafter, stabilization was indicated for the duration of treatment (up to 24 weeks). In mice, total calcium was also exaggerated compared to controls, but there was no difference in total Ca in rats. Serum levels of phosphate essentially remained the same for the duration of treatment for both species studied.

**Fig 4:**
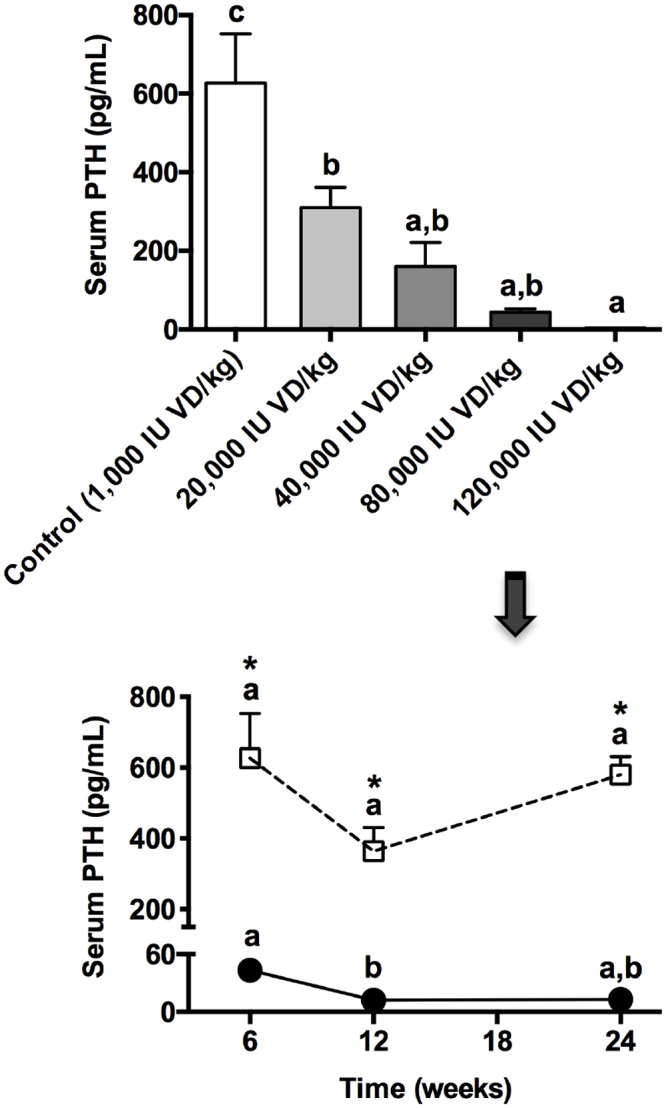
Measures of Parathyroid Hormone (PTH) in vitamin D-supplemented rats. After 12 weeks of intervention, circulating serum concentrations of intact parathyroid hormone (PTH) were determined via commercially available ELISA kits in each of the dietary vitamin D (VD) groups. Data shown as mean ± SEM is indicated in the top graph. Different letters denote statistical significance between groups (p<0.05; n = 8; one-way ANOVA followed by Tukey’s post hoc analysis). Duration-of-treatment effects in each representative group are shown in the bottom frame for either 6, 12 or 24 weeks (control, shown as open squares/dotted line or 80,000IU VD/kg, shown as black circles/solid black line). Different letters are indicative of p<0.05 for duration effects within each group (n = 8; one-way ANOVA) whereas * show p<0.05 compared to respective controls (n = 8; one-way ANOVA). All data is shown as mean ± SEM.

The effect of dietary vitamin D supplementation on circulating PTH concentration in rats is provided in Fig 4. A marked dose response is indicated with serum PTH levels significantly attenuated as a consequence of vitamin D supplementation. Marked suppression of PTH was evident within six weeks of intervention and persistent for the treatment duration of 24 weeks in animals kept on exogenous vitamin D of 80, 000 IU VD/kg. The reported concentration of PTH in rats reported over several decades is variable, indicative of different methods of immunodetection [45, 46]. Nonetheless, vitamin D treatment effects on serum/plasma PTH concentration are as in this study consistently substantive.

**Fig 5:**
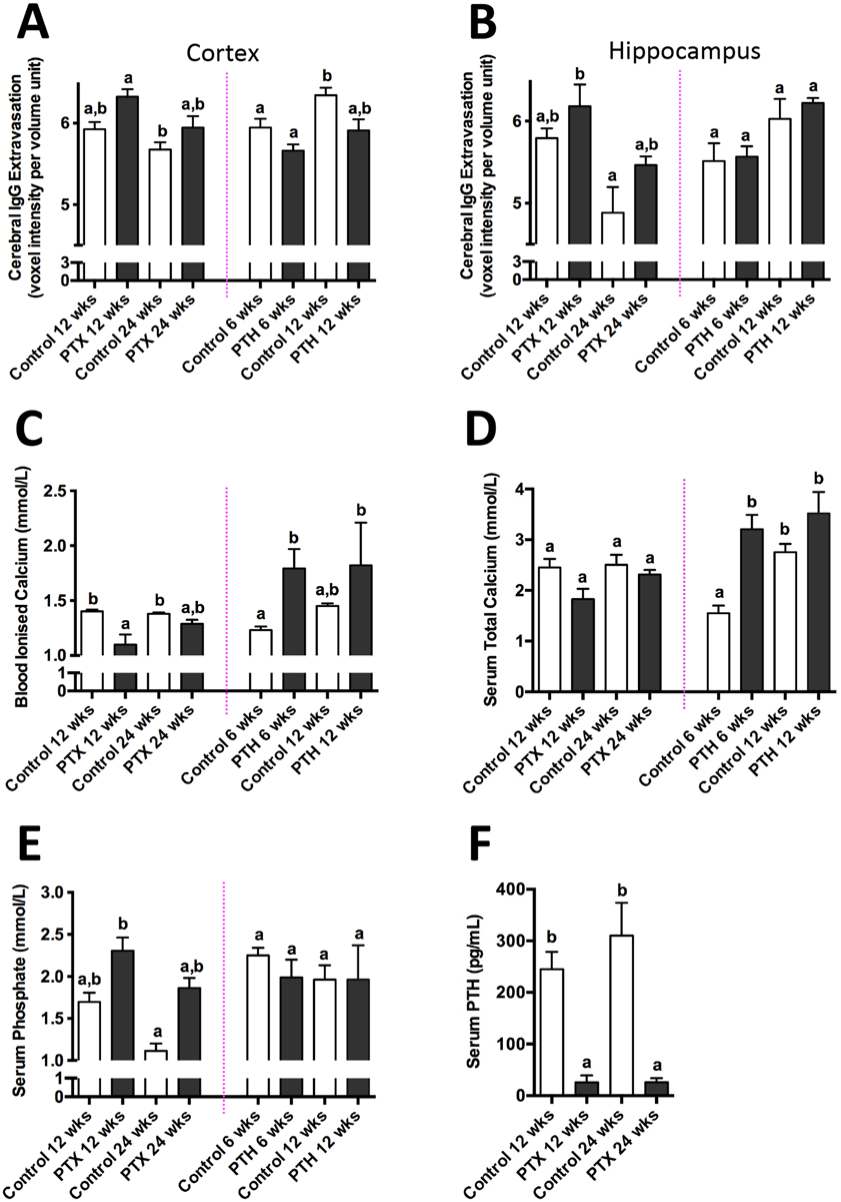
Parathyroidectomy and infusion of Parathyroid Hormone (PTH) studies. Cerebral parenchymal abundance of IgG in the cerebral cortex and hippocampal formation of Sprague-Dawley rats subjected to ablation of parathyroid tissue (PTX) or exogenous infusion of parathyroid hormone (PTH) and their respective controls at either 6, 12 or 24 weeks are shown in graphs A and B. Blood ionised calcium (C), serum total calcium (D), serum phosphate (E) and serum intact PTH (F) were also analysed in each intervention group. Different letters denote statistical significance between groups (p<0.05; n = 4–10; one-way ANOVA followed by Tukey’s post hoc analysis). The data shown are mean ± SEM.

The effects of chronically suppressed serum PTH on capillary permeability without the confounder of dietary supplementation of vitamin D, was explored in other experimental groups, that is in rats with surgical ablation of parathyroid tissue. Fig 5 shows that the parenchymal expression of IgG within CTX and HPF 12 weeks post parathyroidectomy was over 2-fold greater compared to control animals. These effects persisted but were not significantly amplified further when investigated at 24 weeks of intervention. Increased capillary permeability occurred in PTH ablated rats concomitant with a reduction in iCa and to a lesser extent, total Ca. The latter is a contraindication compared to the vitamin D intervention experiments, where increased capillary permeability occurred concomitant with elevated iCa. We note that the serum PTH concentration in control rats for PTH ablation (Fig 5) indicated a lower PTH concentration than in control rats for vitamin D intervention (Fig 4), despite identical serum preparation and measures of PTH being done for all groups simultaneously. With a coefficient of variation for this assay of less than 3%, our interpretation for differences in the concentration of PTH between control groups is the possibility of strain differences in rats hosted at two sites (Charles River, UK for PTH interventions and vitamin D treated groups hosted at Curtin University, Western Australia). Nonetheless, irrespective of this observed difference between the two control group PTH measures, comparison of treatment versus respective control is a valid comparison.

Exogenous provision of PTH via the placement of mini-osmotic pumps predictably increased serum iCa and total Ca. However, consistent with the findings in parathyroidecomized rats, there was no suggestion that capillary permeability had been compromised as a consequence of heightened serum iCa. Rather, parenchymal abundance of IgG within CTX and HPF were comparable between rats given PTH and sham operated control rats hosting osmotic pumps filled only with saline.

Table 2 depicts correlation analysis of cerebral capillary permeability with vitamin D, iCa, total Ca, PTH and phosphate for all vitamin D supplemented and parathyroid-intervention experimental groups. The vitamin D studies in rats and mice suggest that provision of vitamin D and the serum concentration of iCa are strongly associated with increased capillary permeability within CTX and HPF. In addition, the vitamin D supplementation studies also show a marked negative correlation between capillary permeability and the serum concentration of PTH. However, the PTH intervention experiments do not support the contention that the positive association between iCa and capillary integrity indicated in the vitamin D studies is causal. Provision of PTH had markedly increased serum iCa to a comparable degree as dietary vitamin D supplementation, however, PTH treated rats did not demonstrate compromised capillary permeability. Consistent with the findings in PTH treatment of rats, parathyroidectomy-induced disturbances in capillary function also did not support an association of serum iCa with increased capillary permeability. Rather, iCa, total Ca, PTH and cerebral capillary integrity were negatively correlated (or showed no correlation) with parenchymal abundance of IgG. Across all experimental treatment groups, the consistent finding was a negative effect of vitamin D and a positive effect of PTH on capillary integrity.

**Table 2:** Correlation Table of Cerebral Capillary Permeability with markers of Vitamin D-Calcium-Parathyroid hormone homeostasis.

Animals	Intervention	Region of Interest	Vitamin D	Total Calcium	Ionised Calcium	Parathyroid Hormone	Phosphate
**Rats**	**Vitamin D 6 weeks**	**CTX**	0.887**	0.063	0.613**	-0.62**	0.163
		**HPF**	0.803**	0.253	0.679**	-0.68*	0.005
	**Vitamin D 12 weeks**	**CTX**	0.851**	0.619**	0.775**	-0.61**	-0.172
		**HPF**	0.666**	0.529**	0.567**	-0.543**	-0.213
	**Vitamin D 24 weeks**	**CTX**	0.728**	-0.337	0.448	-0.662**	-0.106
		**HPF**	0.534*	-0.174	0.363	-0.349	-0.173
**Mice**	**Vitamin D 6 weeks**	**CTX**	0.300	0.172	0.145	-	-0.164
		**HPF**	-0.191	-0.127	-0.116	-	0.002
	**Vitamin D 12 weeks**	**CTX**	0.768**	0.609**	0.730**	-	-0.291
		**HPF**	0.692**	0.513**	0.613**	-	-0.175
	**Vitamin D 24 weeks**	**CTX**	0.919**	0.565	0.884**	-	-0.106
		**HPF**	0.679*	0.715*	0.765**	-	0.006
**Rats**	**PTX 12 weeks**	**CTX**	-	-0.458	-0.605	-0.608*	0.475
		**HPF**	-	-0.722*	-0.502	-0.079	0.726*
	**PTX 24 weeks**	**CTX**	-	-0.366	-0.629*	-0.228	0.482
		**HPF**	-	-0.674*	-0.099	-0.48	0.325
**Rats**	**PTH Infusion 6 weeks**	**CTX**	-	-0.182	-0.573	-	0.001
		**HPF**	-	-0.051	0.401	-	-0.02
	**PTH Infusion 12 weeks**	**CTX**	-	-0.212	-0.127	-	-0.228
		**HPF**	-	0.208	0.014	-	0.026

Correlation coefficients of cerebral capillary permeability (IgG) in both cerebral cortex (CTX) and hippocampal formation (HPF) with vitamin D, blood ionised calcium, serum total calcium, parathyroid hormone and serum phosphate for all vitamin D supplemented experimental groups (rats and mice), parathyroid gland ablated (PTX) and parathyroid hormone-infused (PTH-infusion) groups are represented in this table. (**p<0.01; *p<0.05; n = 4–10; Pearson’s analysis).

**Fig 6:**
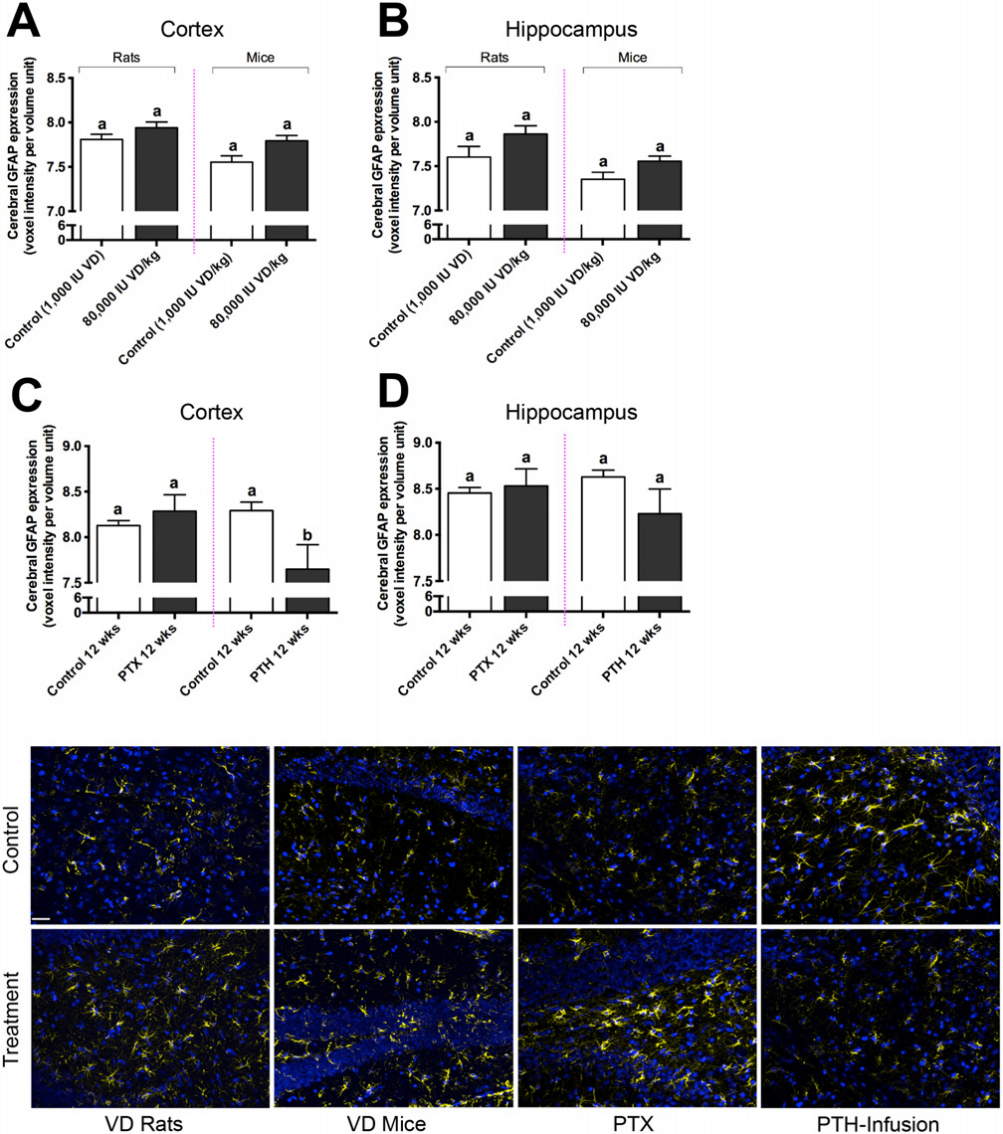
Measure of Neuroinflammation. Neuroinflammation was assessed by measuring the voxel intensity of cerebral glial fibrillary acidic protein (GFAP) expression in cerebral cortex (A) and hippocampal formation (B) in Sprague-Dawley rats and C57BL/6J mice fed 80,000 IU vitamin D (VD) per kilogram of diet and their respective controls, expressed as voxels per volume unit. Cerebral GFAP expression in cortex (C) and hippocampal formation (D) in parathyroid gland ablated (PTX) rats and parathyroid hormone (PTH) infused rats with their controls are also shown. Statistical difference represented by different letters (p<0.05; n = 4–10; one way ANOVA followed by Tukey’s post hoc test). Data is shown as mean ± SEM. Representative 2-dimensional immunofluorescent micrographs of neuroinflammatory GFAP, in extended focus, are shown in the last frame. Each intervention (vitamin D enrichment; PTX; PTH-infusion) and their respective controls are indicated. GFAP is shown in yellow and nuclei in blue. Scale bar indicates 100μm.

Capillary dysfunction characterized by inappropriate extravasation of plasma proteins is postulated to contribute to neurovascular inflammation. Parenchymal abundance of GFAP within CTX and HPF was used as a marker of astroglial activation. Fig 6, demonstrates a modest level of heightened GFAP in rats and mice treated with a supplementary vitamin D regimen that had resulted in increased capillary permeability. In this study, the distribution of parenchymal abundance of IgG was frequently but not consistently associated with GFAP abundance. There was no evidence of an association between parenchymal GFAP with serum levels of iCa, total Ca, serum PTH or with vitamin D treatment per se. Rather, the provision of exogenous PTH to otherwise normal rats was found associated with mild suppression of GFAP activity at 12 weeks in both CTX and HP and conversely, a slight increase in GFAP activity was reported in parathyroidectomized animals.

## Discussion

Increasing evidence suggests that disturbances in blood-brain barrier integrity may increase risk for vascular-based neurodegenerative conditions such as vascular dementia and Alzheimer’s disease. The main objective of this study was to investigate putative regulatory and integrative effects of exogenous vitamin D_3_, calcium and parathyroid hormone on the function of cerebral capillary endothelium and neurovascular inflammation.

Parenchymal abundance of plasma derived IgG was utilized as a surrogate marker of blood-to-brain capillary permeability. Within both the hippocampal formation and cortex of wild-type rats and mice, the findings demonstrate that provision of exogenous dietary vitamin D can significantly compromise the barrier properties of cerebral capillary vessels. Rats appeared to have been more susceptible to the effects of exogenous vitamin D as significant differences compared to control animals were realised within 6 weeks of commencement of the diet, whereas the effect was not realized in mice until 12 weeks of intervention. Furthermore, whilst the concentration of vitamin D incorporated into the diets was equivalent for the two species studied, lower rates of feed consumption relative to body weight, resulted in rats receiving a comparatively lower dose of exogenous vitamin D for each respective treatment arm (Table 1). In both species, the effects of exogenous vitamin D supplementation on parenchymal abundance of IgG were fully realized within 12 weeks of feeding. Thereafter, steady-state levels of parenchymal IgG were indicated within 24 weeks of treatment, suggesting that efflux of parenchymal IgG via either CSF exchange or degradation within epithelial cells of the choroid plexus, may be compensating for blood-to-brain extravasation of some plasma proteins and macromolecules.

Regulation of capillary function or permeability by vitamin D or its bioactive metabolites has not been previously reported, however several lines of evidence suggest the possibility of direct effects on endothelial function. The vitamin D receptor is widely distributed including on vascular endothelial cells, smooth muscle cells, microglia and cerebral neurons [47–50]. It has been demonstrated that 1,25(OH)D_3_ and its precursor 25(OH)D_3_ both directly increase endothelial mediated conversion of vitamin D to the potent metabolite, 1,25(OH)_2_D_3_, by stimulating l-alpha-hydroxylase activity. In rat and human brain endothelial cell cultures, 1,25(OH)_2_D_3_ was reported to increase p-glycoprotein expression and activity [51]. Increased blood-to-brain kinetics of IgG could occur as a consequence of upregulated p-glycoprotein pathway, although this would seem unlikely as it’s considered to be relatively specific. Other potential mechanisms of increased blood-to-brain trans-capillary transport that might be influenced by vitamin D metabolites include modulation of tight junction proteins or adherin expression, or non-specific transcytotic mechanisms. Gascon-Barre and Huet (1983) reported that vitamin D delivery to brain parenchyme is principally free circulating 1,25(OH)_2_D via high affinity binding sites and not associated with plasma levels of 1,25(OH)D per se [52]. If this was the case, then sub-endothelial bioactive metabolites may also influence capillary permeability by modulating pericyte and astrocyte function. However, these alternate regulatory pathways of trans-endothelial transport are yet to be experimentally investigated.

The dose range of exogenous vitamin D_3_ provided to rats and mice in this study was within, or considerably greater than what is ordinarily indicated for clinical therapeutic use due to greater tolerance of vitamin D reported in rodents (Table 1) [53, 54]. Studies suggest that because of differences in l-alpha-hydroxylase activity and in the concentration of chaperone binding proteins, comparably greater amounts of exogenous vitamin D are required to significantly increase serum levels of bioactive vitamin D metabolites [54, 55]. Indeed, clinical studies also demonstrate significant heterogeneity between individuals in serum active vitamin D metabolites with dietary supplementation doses ranging between 1,000 IU to 20, 000 IU VD to increase vitamin D and/or calcium status [56, 57].

Many studies suggest that physiological and toxicological effects of vitamin D are mediated as a consequence of altered cellular loading with iCa. Intracellular iCa is ordinarily orders of magnitude less than in serum, but is generally positively associated with the extracellular iCa concentration. In this study, baseline levels of serum iCa in rats and mice was markedly different, with significantly lower levels in mice maintained on control diets. Provision of dietary vitamin D indicated a dose effect on serum iCa and total Ca, particularly in mice. Similar effects were seen in rats, but the effect was only realized at the higher concentrations of exogenous vitamin D provided. Consistent with the iCa and total Ca data, serum phosphate was reduced in mice with increasing provision of vitamin D, but showed little effect in rats. The relatively weak effects of exogenous vitamin D on iCa is consistent with the species differences indicated and is comparable to the variability reported in humans.

Differences in iCa (rats and mice) and total Ca (mice) compared to control treatment groups were realised within six weeks of commencement of vitamin D intervention and remained relatively constant for up to 24 weeks of feeding. Tai et al reported that acute (15 min changes) in serum calcium did not alter integrity of the BBB indicated by permeability of radiolabelled sucrose, however potential capillary effects with chronically raised serum iCa have not been reported [30]. Clinical studies have however shown a correlation between serum iCa and CSF/serum albumin ratio consistent with direct positive effects on capillary permeability [58]. However, for the latter study caution must be exercised as these subjects had primary hyperparathyroidism. In kidneys, elevated PTH stimulates conversion of calcidiol to the active metabolite (1,25(OH)_2_D_3_). In cell culture studies, direct but paradoxical effects of iCa on endothelial function were demonstrated by Rezic-Muzinic et al who showed the transformation of endothelial cells to a pro-inflammatory phenotype with both sub-optimal and exaggerated exposure to iCa [59]. Parenchymal iCa mediated effects of endothelium, endothelial regulator cells (pericytes/astrocytes) and inflammatory cells (glia) cannot be ruled out. However, in rodent studies maintained on supraphysiological doses of vitamin D, Murphy et al reported that rats had similar levels of CSF and brain extracellular iCa to control rats [31]. The study by Murphy et al suggested that diffusion is the primary modality of trans-endothelial transport of iCa [31]. However, significant regional differences in 45Ca uptake into the CNS have also been reported. The frontal cortex primarily reflects transport across cerebral capillary endothelium, whereas uptake into ventricular CSF reflects transport across the choroid plexuses [30].

In vitamin D supplemented rats and mice, CTX and HPF abundance of IgG was strongly and consistently associated with elevated serum iCa consistent with a causal association. However, this interpretation of the findings was not supported in rats infused with PTH 1–34, where substantial increases in iCa were realized without increased abundance of parenchymal IgG. Similarly, rats with surgical ablation of PTH tissue showed mild increased of IgG expression within CTX and HPF, concomitant with a reduction in iCa. Although it cannot be excluded from these findings, it is unlikely iCa is the primary causative factor in breakdown of capillary endothelium but rather may exacerbate cellular dysfunctioning through promotion of neurotoxic signalling cascades.

A strong negative correlation between capillary permeability and serum levels of PTH was reported in vitamin D supplemented animals and it has been indicated that a causal effect is unlikely to be via regulation of iCa. Whilst it is tempting to suggest the mechanisms may be direct given the substantial ingestion of dietary-derived vitamin D, lower levels of the free active metabolites of vitamin D as a consequence of profound PTH suppression also cannot be excluded.

Direct effects of PTH on capillary function are not reported. However, cell culture studies suggest that epithelial cells possess abundant PTH-receptor 1 and that PTH stimulates vascular endothelial growth factor (VEGF-165), a critical modulator of endothelial cell proliferation [60, 61]. PTH alters the ceramide/sphingosine-1-phosphate rheostat; potentially a key modulator of endothelial function and PTH promotes endothelial nitric-oxide production, a potent vasodilator [61, 62]. Whilst these studies suggest that PTH generally promotes vascular integrity, other reports suggest detrimental physiological effects of exaggerated PTH [34]. However, the latter is indicated only if overloading of cellular iCa is occurring. In this study, infusion of exogenous PTH 1–34 fragment to intact rats maintained on standard chow containing 1,000 IU of vitamin D per kg, increased the serum concentration of iCa to a level comparable to rats maintained on 120, 000 IU vitamin D/kg, but without detrimental effects on cerebral capillary permeability. Therefore, the findings do not support the notion that in this species, hyperparathyroidism directly compromises capillary integrity.

Collectively, capillary permeability appears to be increased in rats and mice maintained on a regimen of vitamin D that is likely to increase active metabolites of vitamin D and suppress PTH synthesis and secretion. The effects appear to plateau, suggesting chronic homeostatic effects. Parenchymal abundance of IgG is used as a surrogate marker of non-specific blood-to-brain protein kinetics, hence presumably inappropriate delivery of other plasma proteins and possibly macromolecules may be occurring. Many studies support the contention that persistent and exaggerated parenchymal abundance of serum derived neurotoxic substances could induce neurovascular inflammation and promote progression of several neurodegenerative disorders. However, in this study, the abundance of GFAP within CTX or HPF was found not to markedly differ in rodent treatment groups of exogenous vitamin D, PTX or PTH treated animals compared to controls. Irrespective of mechanisms, in the rodent species studied the results do not suggest that exaggerated provision of diets enriched in vitamin D significantly promote neurovascular inflammation. However, equally the study design does not investigate if vitamin D per se will attenuate neurovascular inflammation. Previous studies report increased capillary permeability induced by diets enriched in pro-atherogenic lipids and substantial neurovascular inflammation [40, 63, 64]. Clearly, it is a reasonable proposition that excessive vitamin D could have synergistic effects with other nutrients that compromise cerebral capillary integrity. The latter proposition may intuitively seem unlikely given the substantial body of evidence that links low levels of vitamin D with heightened inflammation. However, provision of vitamin D above physiological levels should not be assumed to be beneficial. Indeed, in a cross-sectional study completed as part of a longitudinal clinical study of late-life depression, vitamin D consumption was found to be positively associated with brain lesions in elderly subjects even after controlling for potentially explanatory variables [65]

## Conclusion

The findings from this study reiterate the substantial limitations when considering putative associations of between serum vitamin D, calcium and parathyroid hormone with physiological, pathological or cognitive sequelae. A recent meta-analysis that concluded vitamin D deficiency is associated with a substantially increased risk of all-cause dementia and Alzheimer disease, did not take into consideration an endocrine axis of effects [17]. This study suggests that provision of exogenous vitamin D at levels that suppress PTH secretion and increase iCa concentration compromise the permeability of cerebral capillary vessels but do not promote neurovascular inflammation per se. Potential synergistic effects of vitamin D in heightened inflammatory states should be investigated to further support the putative efficacy of vitamin D supplementation.
